# Sex differences in human atrial electrophysiology: the dark side of the moon? Will engineered heart tissue help to bring light into the darkness?

**DOI:** 10.1007/s00210-025-04587-w

**Published:** 2025-09-17

**Authors:** Djemail Ismaili, Junsoo Im, Renate B. Schnabel, Thomas Eschenhagen, Torsten Christ

**Affiliations:** 1 Institute of Experimental Pharmacology and Toxicology, University Medical Center Hamburg-Eppendorf, Hamburg, Germany; 2 Department of Cardiology, University Heart and Vascular Center Hamburg, Hamburg, Germany; 3 German Centre for Cardiovascular Research (DZHK), Partner Site Hamburg, Kiel, Luebeck, Greifswald, Hamburg, Germany

**Keywords:** Sex differences, Atrial electrophysiology, Atrial fibrillation, Arrhythmia, Engineered heart tissue, Sex hormones

## Abstract

Sex-related differences in cardiac electrophysiology are well established in the ventricle and have reached textbook level. In contrast, our understanding of sex effects in the human atrium remains limited, despite clear clinical observations such as the lower incidence of atrial fibrillation (AF) in women. In this review, we summarize sex differences and discuss potential reasons for the imbalance in knowledge, including the lack of ECG markers for atrial repolarization, the minimal contribution of *I*_Ks_ in atrial tissue, and possible sex differences in inward rectifier currents. We also address the role of aging and hormonal changes, the complexity of studying the perimenopausal transition, and the current limitations of available models. Finally, we highlight the potential of engineered heart tissues to detect genetically encoded differences and to dissect genomic versus non-genomic hormone effects.

## Introduction

The effects of biological sex on biological properties are undisputed. With respect to cardiac chambers, there is a strong disproportion in evidence on mechanisms. Sex-related differences in ventricular physiology and pharmacology have been known for decades and have reached textbook level. The QT interval is longer in females than in males, and female sex itself is an independent risk factor for the development of arrhythmias in both congenital and acquired forms of long QT syndrome (LQTS) (Odening and Koren 2014). Sex steroid hormones are suspected to act as transactivation factors regulating the expression of target genes. However, growing evidence supports the idea of non-transcriptional mechanisms of signal transduction through steroid hormone receptors (Furukawa and Kurokawa 2007). Moreover, sex hormones can modify the dynamic spatiotemporal (regional and transmural) heterogeneities in action potential duration (e.g., the arrhythmogenic substrate) and the susceptibility to autonomic nervous system triggered activity at the tissue, organ, and whole-animal levels (Odening and Koren 2014). The situation in the atrium is in stark contrast (Ravens 2018). The knowledge is much less, despite the fact that from a clinical aspect, sex effects are obvious: women are somewhat protected from the most prevalent arrhythmia in humans, atrial fibrillation (Heeringa et al. 2006; Magnussen et al. 2017; Smith et al. 2025). Mechanisms of sex differences in atrial fibrillation are discussed in two recent excellent reviews (Ko et al. 2016; Odening et al. 2019).

Here, we will discuss some reasons for that disproportion, try to focus on some recent data, and propose some ideas on how to address sex impact on human atrial physiology and pharmacology in the future. Similar to the moon, with advancing methodology and insights, the dark side can increasingly be illuminated. Several central gaps in knowledge currently exist.

## Dark side no. 1: there is no ECG marker of atrial repolarization

**Fig. 1:**
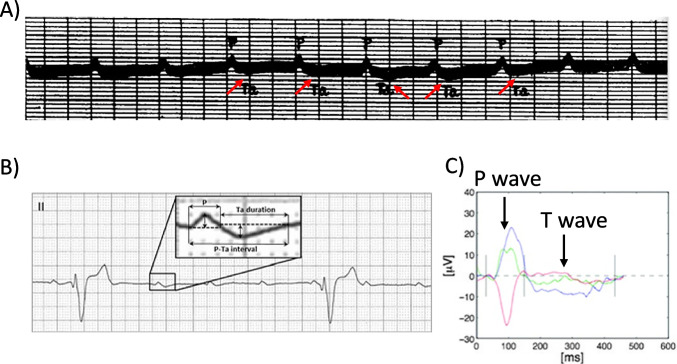
The challenge to detect atrial repolarization wave (“aT” wave).** A**, **B** Atrial T waves unmasked by complete AV block in standard surface ECG. **A** Historic recording in a patient, published in 1925 (reproduced from Sprague and White 1925, public domain). **B** Contemporary recording in a dog (reproduced with permission from Perego et al. 2014). **C** Averaged ECG signals from standard leads I (blue), aVF (green), and V1 (red), recorded by surface ECG (reproduced with permission from Holmqvist et al. 2009). Recordings start with a large P wave. P and T waves indicated by arrows

Bazett developed his first QT correction to heart rate more than a hundred years ago. Analyzing the corrected QT values, he became aware that women had larger values than men. Sample size was rather small (20 men vs. 19 women) (Bazett 1997). However, the absolute value for the difference in QT_c_ between the two sexes found by Bazett (30 ms) was confirmed by many other researchers in large studies (Merri et al. 1989; Rautaharju et al. 1992, 2014; De Bruyne et al. 1999; Bidoggia et al. 2000; Surawicz and Parikh 2002; Vicente et al. 2014). The true nature of the QT interval was not known at that time, since the first action potentials in heart muscle were recorded more than 30 years later (Draper and Weidmann 1951). Nevertheless, it was clear that the T wave marks the end of electrical activity of the ventricle, which begins with the QRS complex, as reported by Einthoven in 1895 (Einthoven 1895). Recording of atrial electrical activity was much more challenging. The first ECG recordings done by Waller were not sensitive enough to detect a wave associated with the electrical activity of the atrium even when recorded in open-chest animals (Waller 1889), because of the much smaller P. ECG recorders quickly improved. However, since even in the ventricle the amplitude of the “ventricular” T wave is much less than the amplitude of the R wave, insufficient sensitivity of ECG recorders was suspected responsible for the failure to detect “atrial T wave.” Several physiologists were able to record very small atrial T waves (following a P wave) when animal heart ventricles were arrested by drastic interventions, e.g., by simple dissection. In 1912, Hering called the recorded wave “aT” (atrial T wave). Probably more importantly, Hering could definitely identify the reason for the ongoing difficulties to record atrial T waves: the temporal coincidence of the very small aT and the large R wave from ventricles (Hering 1912). Thus, medical doctors had to wait for patients who survived complete heart block to scrutinize the aT wave. More than 10 years later, five such cases were reported (see Fig. 1) (Sprague and White 1925). The PaT duration recorded in this study was close to action potential duration measured later in human atrial tissue in vivo (Franz et al. 1997) and in vitro (Escande et al. 1985; Pecha et al. 2023). The issue of aT being superimposed by R remains, even when sophisticated signal-averaging methods were applied. Authors stated in 2009: “Although the aT peak may occasionally be located in the PQ interval during normal AV conduction, it is unlikely that enough information can be obtained from analysis of this segment to differentiate normal from abnormal atrial repolarization. Hence, an algorithm for QRST cancellation during sinus rhythm is needed to further improve analysis” (Holmqvist et al. 2009). It remains to be seen if newer techniques like artificial intelligence will be helpful (Schuijt et al. 2024).

## Dark side no. 2: in the human heart, the link between sex and repolarization in the ventricle is less important in the atrium

Longer QT_c_ and ventricular APD_90_ in females than in males predict a difference within the delicate relationship between depolarizing and repolarizing forces. The classic and most prevalent type 1 long-QT (LQT1) syndrome is caused by a loss of function of the slowly activating delayed rectifier current *I*_Ks_ (Moss et al. 2007). Women respond to HERG (*I*_Kr_) block with a larger increase in QT (like LQT1 patients), a finding that could be explained by lower *I*_Ks_ in women (Drici et al. 1996). However, despite the fact that loss of *I*_Ks_ as seen in LQT1 syndrome is associated with marked QTc prolongation, it remains a challenge to demonstrate *I*_Ks_ contribution in ventricular tissue of several species (including man) under in vitro conditions (Christ et al. 2015). Concomitant block of the other repolarizing potassium current, the rapidly activating delayed rectifier current *I*_Kr_, introduced as “reduced repolarization reserve,” was the seminal step to unmask *I*_Ks_ contribution to APD_90_ in ventricles of dog (Biliczki et al. 2002) and guinea pig (Schuijt et al. 2024). In dog heart, stimulation of β-adrenoceptors is an alternative approach to unmask *I*_Ks_ contribution to APD (Volders et al. 2003). The situation in human ventricle is more complex since both interventions (stimulation of β-adrenoceptors and *I*_Kr_ block) are needed to uncover *I*_Ks_ contribution to APD (Jost et al. 2005, 2013; Lemoine et al. 2018). From the findings in human ventricles, it may not come as a surprise that simple block of *I*_Ks_ does not prolong APD in human atrial tissue. Again, concomitant potassium channel block (either *I*_Kr_ or the ultra-rapid activating delayed rectifier potassium current *I*_Kur_) was ineffective to unmask *I*_Ks_ contribution to APD. In contrast to ventricles, *I*_Ks_ cannot be increased by β-adrenoceptor stimulation in human atrial cardiomyocytes (Sönmez et al. 2024). Thus, we would expect even less *I*_Ks_ contribution in the human atrium vs. ventricle. This conclusion is supported by clinical findings (Johnson et al. 2008). However, there are distinct differences between LQTS subtypes in subjects younger than 60 years. While the risk for AF was clearly higher in LQT3, it was lower in LQT2 and not changed in LQT1 (Platonov et al. 2019). Importantly, other currents than *I*_*Ks*_, mainly *I*_*Kr*_, *I*_*K1*_, and *I*_*Ca,L*_ are involved in sex differences of human atrial electrophysiology (Furukawa and Kurokawa 2007). Estrogen, progesterone, and testosterone have partially opposite effects on *I*_*Ca,L*_, *I*_*Ks*_, and *I*_*Kr*_, depending on concentration of hormones and animal models used (Prajapati et al. 2022). Therefore, effects of sex hormones on repolarization reserve are hard to predict.

## Dark side no. 3: are inward rectifier currents smaller in female vs. male hearts?

**Fig. 2:**
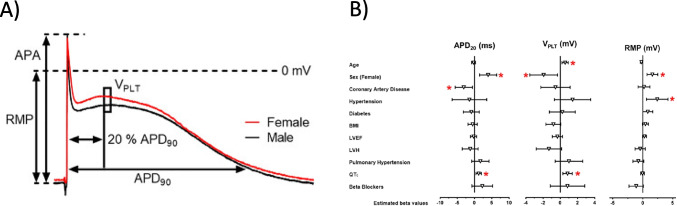
Electrophysiological parameters in human atrial tissue from patients in sinus rhythm.** A** Representative action potentials recorded from right atrial trabeculae of a female (red) and a male (black) patient (mean age = 67 years) in sinus rhythm. Action potential parameters as illustrated: action potential amplitude (APA), resting membrane potential (RMP), action potential duration at 20%, 50%, and 90% repolarization (APD_20_, APD_50_, APD_90_), and plateau potential (V_PLT_), defined as the mean membrane potential 5 ms after APD_20_. **B** Forest plot showing estimated effect sizes of selected clinical variables on APD_20_, V_PLT_, and RMP based on a multivariable regression model. Both panels reproduced with permission from Pecha et al. 2023

In the past, we have measured action potentials in atrial tissues of several hundred patients in search of new atrial-selective antiarrhythmics (Fig. 2). This unique data set is large enough to scrutinize for sex differences. And in fact, even in a first, rather preliminary analysis, sex differences could be detected. RMP was less negative in preparations from females, associated with lower V_max_ and APA (Ravens 2018). These results could be confirmed in a more sophisticated re-analysis using multivariable analysis (Pecha et al. 2023). Interestingly, there was no association between QTc and atrial APD_90_, supporting the assumption that sex differences of repolarization in the ventricle are not simply mirrored in the atrium. The finding that longer QTc associates with longer plateau phase (APD_20_) in female atria could reinitiate speculations on whether *I*_Ca,L_ could be involved in sex differences in cardiac electrophysiology, which links sex differences in *I*_Ca,L_ to hormone-driven changes in expression and current density. *I*_Ca,L_ is larger in human and dog female than male heart, but does not differ between the two sexes in hearts from smaller animals like mice and rat (for excellent review, see Prajapati et al. 2022). The difference in RMP between atrial tissues from men vs. women was unexpected. Weaker inward rectifier currents could explain the finding. Such a speculation is supported by the finding that the difference in RMP is lost in chronic atrial fibrillation, a pathology where inward rectifiers undergo substantial remodeling (Dobrev et al. 2001, 2005; Balana et al. 2003). We would even go one step further and speculate on whether lower inward rectifier currents in females could be a general finding in the human heart. *I*_K1_ was found smaller in some studies in ventricular cardiomyocytes from female versus male animals for both rabbits (Liu et al. 1998) and guinea pigs (James et al. 2004) (for extensive review, see Prajapati et al. 2022). No data on sex differences in *I*_K1_ or RMP are available from human ventricular cardiomyocytes or tissues. However, expression of Kir2.3 is markedly lower (~ 50%) in ventricular tissue from healthy females vs. males (Gaborit et al. 2010). Anyhow, we feel further work is warranted to explore a potential role of inward rectifier currents in sex-related differences of human cardiac electrophysiology in both atrial and ventricular models. From the finding that atrial *I*_K1_ was larger (and not smaller!) in female rabbits at a young age, one could expect that this may have a large impact on sex-dependent differences in atrial electrophysiology (see below) (Giammarino et al. 2025).

## Dark side no. 4: sex differences in the elderly: what could be the mechanistic links for the sex-related AF-risk in aged persons?

**Fig. 3:**
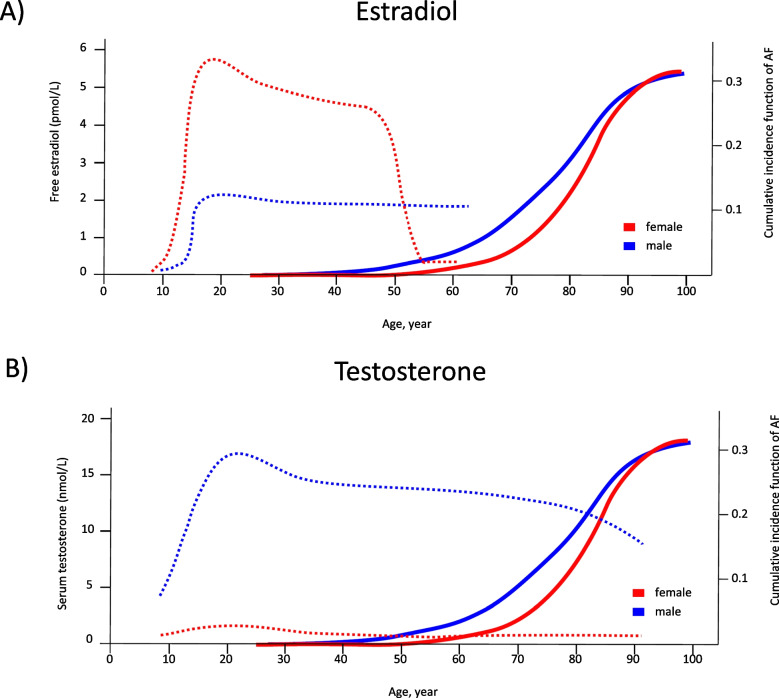
Hormone levels and AF incidence across age and sex. Free estradiol (**A**) and serum testosterone concentrations (**B**) plotted as dotted lines, and cumulative incidence of atrial fibrillation (solid line), across the lifespan in females (red) and males (blue). Curves were redrawn from Frederiksen et al. 2020, Handelsman et al. 2016, and Magnussen et al. 2017

There is a general interest to understand how sex hormones may affect cardiac electrophysiology. Shortening of the QT_c_ in males with the onset of puberty (Rautaharju et al. 1992) suggests genomic effects of sex hormones. Such an assumption is supported by the fact that ion currents measured in sexually mature female animals fluctuate following the ovarian cycle(James et al. 2004). Sex hormones can affect ion channels directly (for excellent and extensive review, see Prajapati et al. 2022). From clinical studies, it is known that the incidence of AF rises when the level of sex hormones falls (Handelsman et al. 2016; Magnussen et al. 2017; Frederiksen et al. 2020). One could simply argue that the AF-protective action of sex hormones is lost. Obviously, the situation is more complex. In ageing men, who are more affected by AF than women, the drop in testosterone levels is rather modest compared with the sharp decline in estrogen levels observed in ageing women (Fig. 3). On the other hand, the effect of testosterone on AF risk follows a U-shaped curve as reviewed recently (Mason et al. 2025). Furthermore, testosterone in females and estrogen in males remain low throughout the lifespan (Handelsman et al. 2016; Frederiksen et al. 2020), arguing against the idea that decreases in sex hormones promote AF per se.

It appears that maintaining a proper balance between sex hormones could be an important factor. While estradiol drops sharply in postmenopausal women, progesterone concentrations decrease significantly after menopause, but less strongly and without rhythmic fluctuation (Henderson 2018), leading to an imbalance between female sex hormones. Clinical studies showed that the AF risk was increased when estradiol was applied alone, but decreased when estradiol was combined with progestin (a compound that activates progesterone receptors) (Lee et al. 2021). This clinical finding may highlight the importance of hormonal balance and suggests that progesterone, as well, may play a role in atrial electrophysiology.

In males, estrogen concentrations are physiologically lower than in females (Frederiksen et al. 2020). Aromatase in non-gonadal tissue, including pericardial fat (Bernasochi et al. 2017), converts testosterone into estrogen. Since testosterone declines at higher ages, the substrate for aromatase drops. In addition, there is a decline in aromatase activity, potentially restricting local sex hormone effects.

There could be another link: the stronger resistance against AF in women could be genetically encoded. Although men and women share nearly identical genomes apart from sex chromosomes, increasing evidence suggests that sex-specific electrophysiological traits may have a genetic component (Gaborit et al. 2010; Øyen et al. 2012; Ambrosi et al. 2013). A recent finding supports such an assumption. In young, pubertal rabbits, the RMP in female atrial cardiomyocytes was more negative than in males (Giammarino et al. 2025). This sex difference in RMP occurred at very low estradiol levels (comparable to those in ovariectomized females(Odening et al. 2012), suggesting a hormone-independent, possibly genetic, origin. On the other hand, testosterone is able to reduce *I*_K1_ and could thereby mimic sex differences in female cardiomyocytes (Giammarino et al. 2025). Recent transcriptomic data from human sinus node tissue revealed sex-associated differences in the expression of pacemaker-related genes, potentially contributing to the higher resting heart rate and increased tachycardia susceptibility observed in females (Li et al. 2025). While transcriptomes are shaped by multiple factors, including hormonal influences, these findings are at least consistent with the hypothesis of intrinsic, possibly genetically modulated, sex differences in cardiac electrophysiology.

## Dark side no. 5: what could be the appropriate models to study sex-related differences in the electrophysiology of the ageing human atrium?

**Fig. 4:**
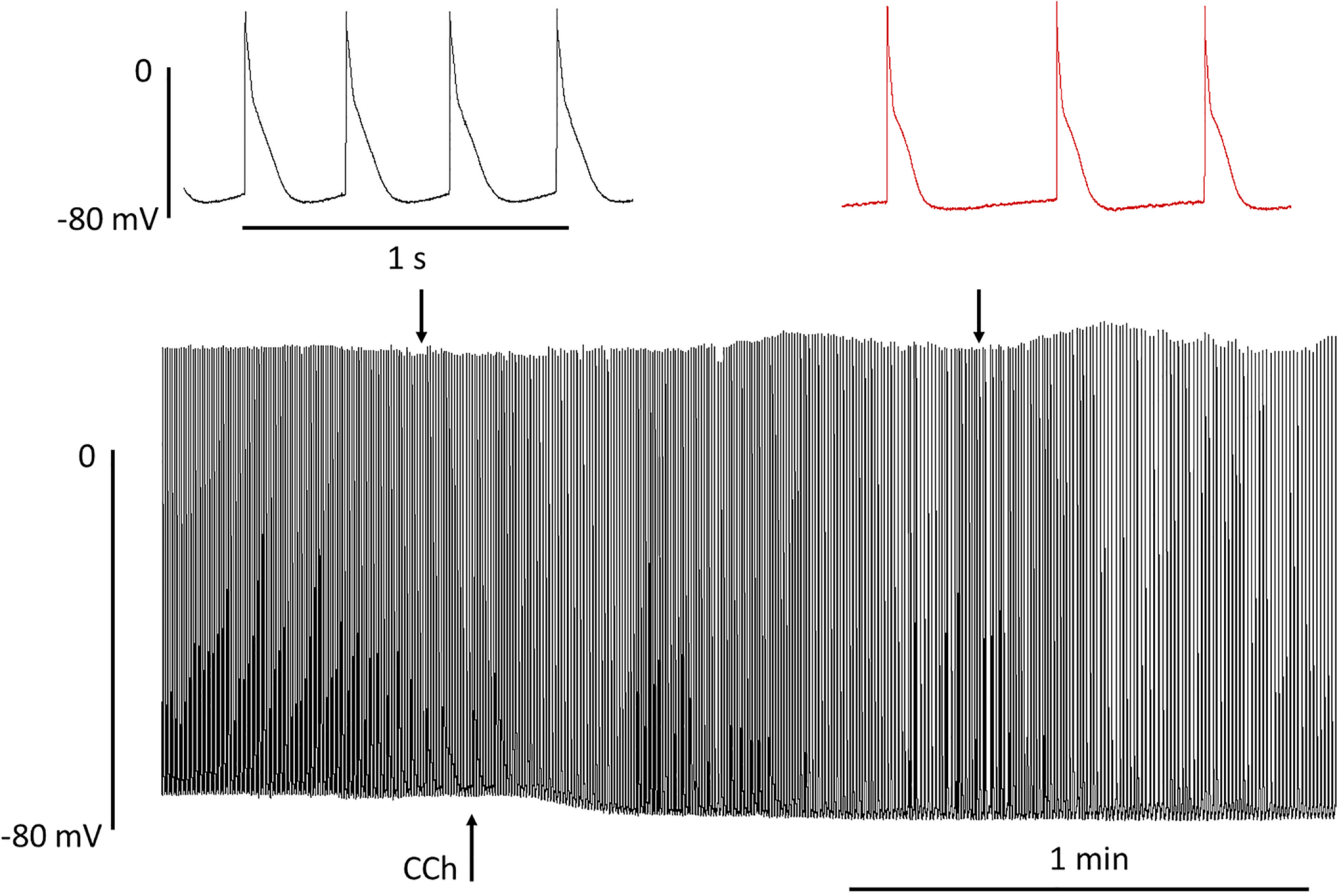
Effect of muscarinic receptor activation on spontaneously beating atrial EHT. AP recorded at low speed in an aEHT exposed to 10 µM carbachol (CCh) indicated by an arrow. Please note the drop in maximum diastolic potential. Top: original registrations recorded at time points indicated by arrows. Same scaling for both. Please note slowing of beating rate in the presence of CCh

Atrial tissue can be collected during open heart surgery and is instrumental in improving our understanding of atrial pathophysiology in AF (Christ et al. 2014; Greiser et al. 2014). If sample sizes are large enough to allow multivariable analysis, such studies can be used to scrutinize sex effects (Pecha et al. 2018, 2020). Patients undergoing open heart surgery in the western world are generally 60 years or older. Younger patients, in particular, premenopausal, are hard to find, making any observation rather static, precluding the study of the transition phase to menopause that is associated with the steep increase in AF. Animal studies have been used in the past to study sex hormone effects on ventricular electrophysiology. However, there are substantial species differences in cardiac electrophysiology (Schotten et al. 2011; Clauss et al. 2019). Effects of sex hormone application are complicated by intrinsic hormone production that cannot be eliminated easily. Engineered heart tissues (EHT), three-dimensional cardiac constructs derived from human-induced pluripotent stem cell–derived cardiomyocytes, can be cultured in the absence of any sex hormones, enabling the detection of genetically encoded differences in atrial electrophysiology. Short- vs. long-time (some minutes vs. some day) exposure to sex hormones should allow dissection of non-genomic vs. genomic effects. Atrial EHT recapitulates key findings of action potential regulation in the human adult atrium (Lemme et al. 2018; Schulz et al. 2023). Early repolarization is dominated by the atrial-selective *I*_Kur_ (Schulz et al. 2023). *I*_Kr_ dominates over *I*_Ks_ in the regulation of late repolarization (Sönmez et al. 2024). Activation of muscarinic receptors hyperpolarizes diastolic potentials, shortens APD, and slows beating (Fig. 4). It is open whether the spontaneous beating rate of atrial EHT and its regulation can provide meaningful insight into sex-related differences in heart rate regulation (Ramaekers 1998).

There is a concern about the immaturity of hiPSC-CM, potentially limiting translation to the situation in humans. Specifically, the application of retinoic acid does not necessarily produce an action potential shape like that of the human atrium (Schulz et al. 2024). Nevertheless, we are confident that it is worth investigating whether atrial EHT could help to understand sex hormone effects better. It remains to be elucidated whether the accuracy of the electrophysiological experiment using atrial EHT is strong enough to detect genomic effects of sex hormones.

## Data Availability

All source data for this work (or generated in this study) are available upon reasonable request.

## Abbreviations

AF: Atrial fibrillation
AP: Action potential
APD: Action potential duration
AV: Atrioventricular
ECG: Electrocardiogram
EHT: Engineered heart tissue
I_Ca, L_: L-type calcium current
I_K1_: Inward rectifier potassium current
I_Ks_: Slowly activating delayed rectifier potassium current
I_Kr_: Rapidly activating delayed rectifier potassium current
I_Kur_: Ultra-rapid delayed rectifier potassium current
RMP: Resting membrane potential
